# No Difference in Bone Tunnel Enlargement and Clinical Outcome between Cortical Suspension and Hybrid Femoral Fixation in Hamstring Anterior Cruciate Ligament Reconstruction

**DOI:** 10.1111/os.14024

**Published:** 2024-03-06

**Authors:** Yucheng Lin, Lu Zhang, Sinuo Shen, Yuzhi Chen, Li Xu, Mingliang Ji, Yudong Guo, Jinan Wei, Yonggang Li, Xiaotao Wu, Jun Lu

**Affiliations:** ^1^ Department of Orthopaedic Surgery Zhongda Hospital, School of Medicine, Southeast University Nanjing China; ^2^ Department of Anesthesiology Women's Hospital of Nanjing Medical University, Nanjing Maternity and Child Health Care Hospital Nanjing China

**Keywords:** anterior cruciate ligament reconstruction, bone tunnel enlargement, clinical outcome, cortical suspension fixation, hybrid femoral fixation

## Abstract

**Objective:**

The best method for femoral fixation in anterior cruciate ligament reconstruction (ACLR) remains controversial. The study assesses the bone tunnel enlargement and clinical outcome in hamstring ACLR using cortical suspension or hybrid (cortical suspension and compression) femoral fixation.

**Methods:**

From January 2010 to December 2021, 102 patients who underwent quadruple hamstring ACLR using cortical suspension (39 patients) or hybrid (63 patients) fixation on the femoral side were retrospectively analyzed. Clinical evaluation was conducted using the international knee documentation committee score, the Lysholm score, the Tegner activity level scale, the knee injury and osteoarthritis outcome score (quality of life score), the Lachman test, and the side‐to‐side difference by the KT‐1000 arthrometer. The complications after the surgery were also evaluated. These data were compared at baseline and last follow‐up. The diameters of the femoral tunnel were calculated at three sites: the width of the entrance of the femoral tunnel, 1 cm proximal to the entrance of the femoral tunnel and the largest diameter of the femoral tunnel on magnetic resonance imaging (MRI) coronal images. Bone tunnel widening data were contrasted between MRI images conducted at least 2 years and within 2 weeks after surgery. The morphology of bone tunnel enlargement was also observed and recorded. The categorical parameters were analyzed using the χ^2^‐test and Fisher's exact test. The continuous variables conforming to a normal distribution were analyzed using Student's *t*‐test, and the Mann–Whitney *U*‐test was undertaken between the two groups without normal distribution.

**Results:**

Both cortical suspension and hybrid femoral fixation in quadruple hamstring ACLR achieved significantly improved patient‐reported outcome scores and knee stability compared to preoperative data. However, no significant differences were found between these two methods in clinical evaluations, postoperative complications, and patient‐reported outcome scores. Although the mean diameter of the enlarged bone tunnel was lowered by an additional bioabsorbable interference screw fixation near the joint line, a statistically insignificant difference was found between the hybrid and cortical suspension fixation on the femoral side. There was no statistical difference in the distribution of enlarged bone tunnel morphology between groups.

**Conclusions:**

No significant difference was found in the bone tunnel enlargement and clinical outcome between cortical suspension and hybrid femoral fixation in ACLR using hamstring autograft.

## Graphical abstract

Both cortical suspension and hybrid femoral fixation techniques in hamstring anterior cruciate ligament reconstruction significantly improve postoperative outcomes, with no significant differences between the two methods in terms of bone tunnel enlargement, clinical evaluations, postoperative complications, and patient‐reported outcome scores.

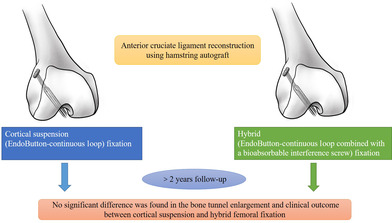

## Introduction

Anterior cruciate ligament (ACL) rupture mostly affects young patients globally.^1^ ACL reconstruction (ACLR) is the currently preferred surgical treatment method for young ACL injuries.^2^ It is common knowledge that integration of the ACL tendon graft into the bone tunnels is presumed to be a key element to the success of the procedure.^3^ However, despite the development of surgical techniques, the process of tendon–bone healing remains inadequate.^4^ The integration of bone tunnels and ACL tendon grafts often leads to “bone tunnel enlargement” (BTE), which has been linked to poor tendon–bone healing in previous studies.^5^, ^6^, ^7^ The mechanisms of this tunnel enlargement remain unclear but are presumed to be multifactorial, with mechanical and biological factors.^8^, ^9^ Among the multiple factors, the type of tunnel fixation on the femoral side seems to be an important issue.^6^ Intriguingly, the type of femoral fixation in ACLR with hamstring autograft directly identifies the graft motion in the bone tunnel and even affects synovial fluid leakage and the release of cytokines.^10^ Furthermore, there are mixed and contrasting opinions regarding the optimal femoral fixation method for hamstring tendon autograft in ACLR. Currently, there is no study indicating a superior method of femoral fixation.

Common techniques for femoral fixation in hamstring ACLR are indirect cortical suspensory fixation (e.g., EndoButton‐Continuous Loop, Smith & Nephew Endoscopy, Andover, MA) and direct compression fixation (e.g., BIOSURE, Bioabsorbable Interference Screw, Smith & Nephew Endoscopy, Andover, MA). Fixed‐length EndoButton devices are typically used on the femoral side with hamstring grafts and have been demonstrated to offer excellent biomechanical stability and generally good clinical outcomes with a low failure rate.^11^, ^12^ However, the application of such suspensory fixation devices might influence both the bungee cord and windshield wiper effects by allowing excessive motion of the graft, which will induce BTE.^6^, ^13^ In recent years, second‐generation adjustable‐loop devices were introduced (e.g., TightRope, Arthrex, Naples, FL). Their loop can be tightened and adjusted according to the tunnel length during the surgical process, thereby decreasing the possibility of a bungee cord effect.^14^ Although the adjustable mechanism is superior in design, some studies have pointed out that the loop will loosen and lengthen during cyclic loads, creating graft laxity.^15^ Furthermore, biomechanical studies have shown a considerable amount of loosening in adjustable‐loop devices, which might affect clinical results after ACLR.^16^, ^17^ The application of a bioabsorbable interference screw allows the graft to be strongly fixed at the level of the joint line, which will help minimize BTE.^18^ In contrast, fixation with such a screw might facilitate osteoclastic activity and cytokine release and also enlarge the tunnels during screw insertion.^19^, ^20^ Given the lack of an optimal choice for the type of femoral bone tunnel fixation in ACLR, a hybrid fixation that combines the advantages of both methods was proposed.^21^ The available body of literature suggests a lack of sufficient evidence to recommend the use of cortical suspension only or together with the compression fixation devices on the femoral side in ACLR.

We designed this study to find an optimal femoral fixation method in the hope of achieving the least BTE and better clinical results. The purpose of this study was: (1) to investigate the BTE of patients undergoing hamstring ACLR using cortical suspension only or hybrid (cortical suspension and compression) femoral fixation; and (2) to compare the clinical outcome of the two distinct femoral fixation methods in ACLR.

## Methods and Materials

### Basic Information

Between January 2010 and December 2021, at a single institution, 301 patients who underwent ACLR with quadruple hamstring autograft using cortical suspension or hybrid (cortical suspension and compression) fixation on the femoral side and the bioabsorbable interference screw fixation on the tibial side were retrospectively analyzed. A retrospective evaluation protocol was established and authorized by the institutional ethics review board (2021ZDSYLL345‐P01).

The inclusion criteria were as follows: (i) ACL rupture due to a clear history of knee trauma; (ii) patients over 18 years of age; (iii) patients undergoing ACLR surgery using quadruple hamstring autograft; and (iv) the femoral side graft was fixed with EndoButton‐Continuous Loop or EndoButton‐Continuous Loop combined with a bioabsorbable interference screw, and the tibial side was fixed with a bioabsorbable interference screw (Smith & Nephew Endoscopy, Andover, MA, USA).

The exclusion criteria included: (i) multi‐ligamentous injury; (ii) conduct of a revision procedure; (iii) a history of previous contralateral knee ligamentous injury; (iv) reoperation of the ipsilateral knee after surgery; (v) Kellgren–Lawrence grade >2 on preoperative knee X‐rays; (vi) intraoperative arthroscopic observation of cartilage defect; and (vii) patients who could not be contacted or were lost to follow‐up.

**FIGURE 1:**
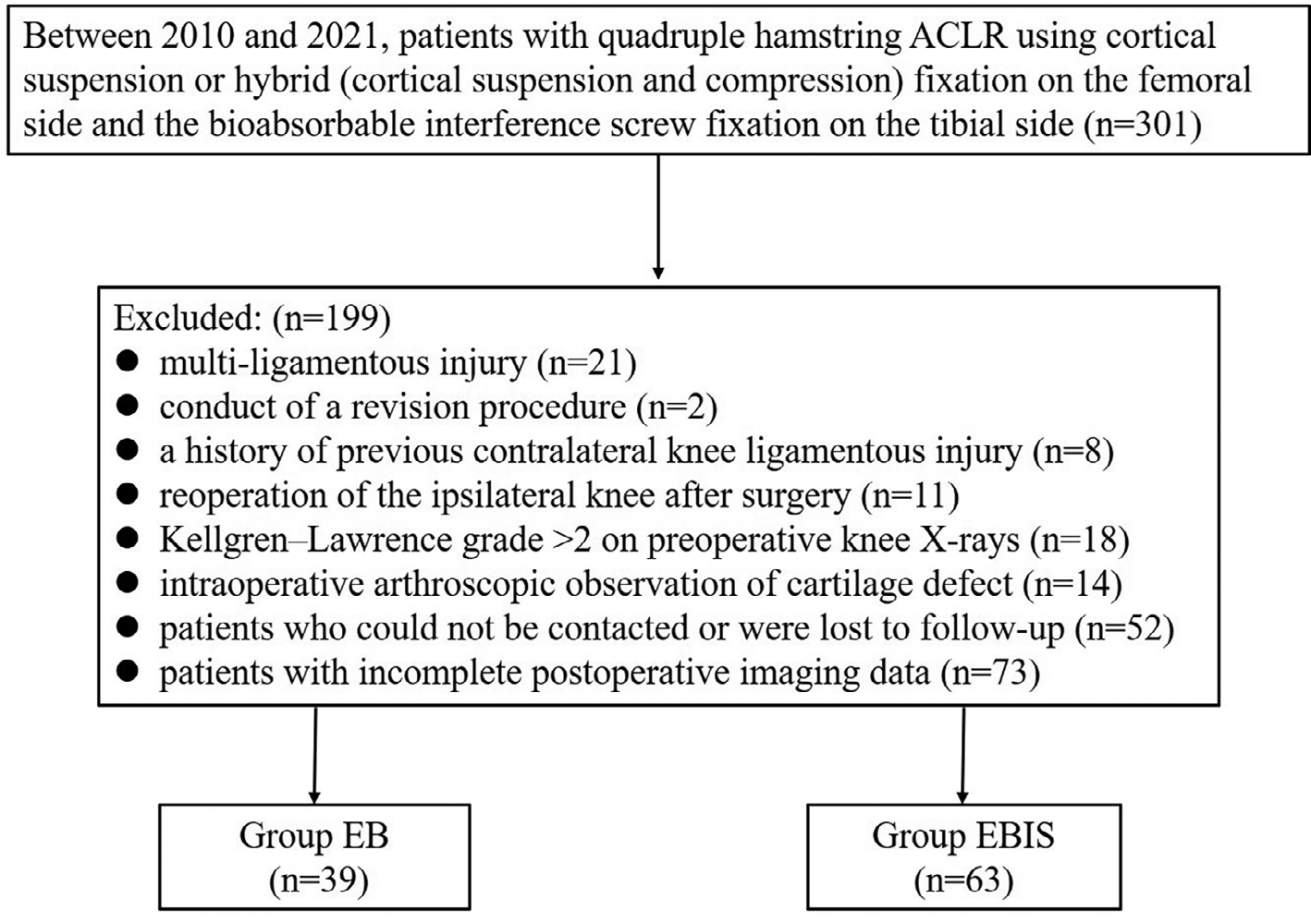
Patient selection flowchart. ACLR, Anterior cruciate ligament reconstruction; EB, EndoButton‐Continuous Loop; EBIS, EndoButton‐Continuous Loop combined with a bioabsorbable interference screw.

**FIGURE 2:**
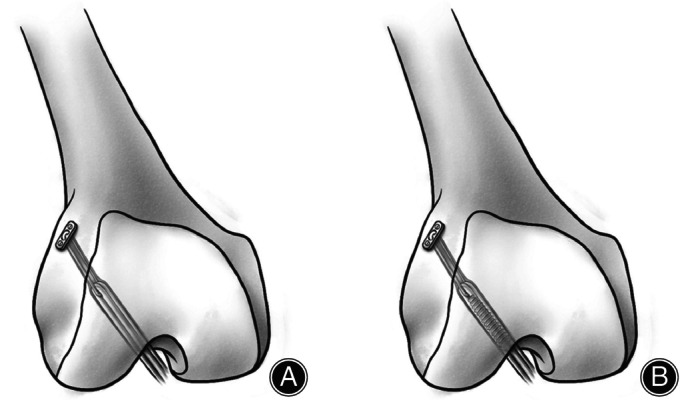
Schematic diagram of the different fixation methods of the femoral tunnel. (A) cortical suspension (EndoButton‐Continuous Loop) fixation (Group EB), (B) hybrid (EndoButton‐Continuous Loop combined with a bioabsorbable interference screw) fixation (Group EBIS). EB, EndoButton‐Continuous Loop; EBIS, EndoButton‐Continuous Loop combined with a bioabsorbable interference screw.

The study eventually included 102 patients (Figure 1). Two groups were outlined according to the fixation method used on the femoral side (Group EB [39 patients, cortical suspension fixation used EndoButton‐Continuous Loop, Smith & Nephew Endoscopy, Andover, MA]; Group EBIS [63 patients, hybrid fixation used EndoButton‐Continuous Loop combined with a bioabsorbable interference screw, Smith & Nephew Endoscopy, Andover, MA]) (Figure 2). All patients included in this study underwent a clinical follow‐up over 2 years, with accompanying radiological follow‐up data.

### Surgical Technique

A skin incision measuring approximately 4 cm was made on the anteromedial tibial surface. The semitendinosus, as well as gracilis tendons, were then harvested from their distal insertion. The graft was folded twice to make it four‐stranded and attain a proper length of approximately 85–95 mm. The diameter of the hamstring graft ranged from 7 to 9 mm. The axilla of the graft was placed via the loop of the EndoButton‐Continuous Loop (Smith & Nephew Endoscopy, Andover, MA).

Before reconstruction, meniscal tears were repaired, and irreparable tears were treated with partial meniscectomy for patients with meniscal issues. Once the preparation was complete, we began to develop the femoral tunnel. Under maximal knee flexion on the table, we drilled the femoral tunnel through the accessory medial portal with an inside‐out method. The femoral socket was positioned at 10 o'clock for the right knees and 2 o'clock for the left knees.^22^ For the tibial tunnel, the transtibial technique was used. The tibial tunnel was shifted to match the diameter of the graft by using reamers that were compatible with the size of the grafts.

In group EB, the graft was inserted through the tibial tunnel and into the femoral tunnel using the “lead” suture on the EndoButton‐Continuous Loop. After the graft was totally seated in the femoral tunnel, the “follow” suture was pulled to flip the EndoButton. In the EBIS group, after the graft was fully seated in the femoral tunnel and the EndoButton was flipped, a bioabsorbable interference screw (6 mm in diameter and 20 mm in length; BIOSURE, Bioabsorbable Interference Screw, Smith & Nephew Endoscopy, Andover, MA) was conducted in the femoral socket for strengthening fixation. The length of the introduction of the graft into the femoral tunnel was 20 mm in both group EB and group EBIS. Following 20 sets of flexion and extension, the tibial side graft was secured with a bioabsorbable interference screw (BIOSURE, Bioabsorbable Interference Screw, Smith & Nephew Endoscopy, Andover, MA) in the tibial tunnel for patients in both groups. The diameter of the tibial screw was equivalent to that of the tibial bone tunnel.

### Rehabilitation

All patients received the same rehabilitation protocol. Quadriceps and active range of motion (ROM) exercises in sitting or lying positions without weight‐bearing were begun as soon as possible. An ice bag was applied to the operative knee for 20 min, three times a day, during the first postoperative week. Weight‐bearing training was also implemented with the assistance of a knee brace (starting from full extension and flexed 20°weekly thereafter) for 6 weeks. High‐demand pivoting sports activities were authorized after 6–9 months.

### Assessment of Clinical Outcome

At a minimum of 2 years postoperatively, the included patients were called for an assessment. The time points for postoperative follow‐up were July 1, 2023 and November 26, 2023. Objective clinical examinations included the Lachman test and the side‐to‐side difference using the KT‐1000 arthrometer (MEDmetric, SanDiego, CA). We followed the protocol to conduct the KT‐1000 test.^23^ Briefly, tibial translation was calculated in millimeters at an anterior pulling force of 134 N and with the knee in a flexed position at 30°. A side‐to‐side difference between the ACL‐reconstructed knee and the contralateral healthy knee was computed. Patient‐reported subjective scores, including the International Knee Documentation Committee (IKDC) score,^24^ the Lysholm knee score,^25^ the Tegner activity level scale,^26^ and the knee injury and osteoarthritis outcome score^27^ (KOOS) ‐ quality of life scale were also computed. The complications after the surgery, such as deep vein thrombosis, secondary meniscal lesions, knee stiffness, anterior knee pain, graft failure requiring revision surgery, and contralateral ACL injury, were evaluated.

### Radiological Evaluation

**FIGURE 3:**
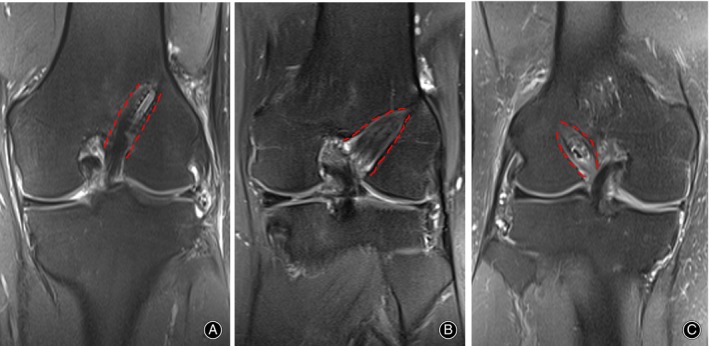
MRI images showing the femoral tunnel morphology on coronal views. (A) Line type in EBIS group, (B) cone type in EB group, and (C) fusiform type in EBIS group. EB, EndoButton‐Continuous Loop; EBIS, EndoButton‐Continuous Loop combined with a bioabsorbable interference screw.

Magnetic resonance imaging (MRI) (scanning series: repetition time/echo time, 2800/33 ms; voxel size: 0.8*0.6*3.0 mm; slice thickness: 3.0 mm; Siemens MAGENETOM Trio, 3.0 T, Germany) was conducted, respectively, within 2 weeks after surgery and at least 2 years after surgery in patients. All radiographic measurements were executed by two blinded observers using the automatic distance measurement tool of the Picture Archiving and Communicating System (Neusoft, China). Tunnel sizes were identified by measuring the width of the entrance of the femoral tunnel, the bone tunnel perpendicular to the long axis of the tunnel 1 cm from the joint line and the largest diameter of the femoral tunnel on coronal images. Measurement of BTE was estimated as follows: Enlargement of the bone tunnel (mm) = MRI measured diameters (at least 2 years after surgery) − MRI measured diameters (within 2 weeks after surgery). We also assessed and recorded the morphology of BTE (Figure 3). We classified enlarged bone tunnel morphology into three types^28^: (i) line type, (ii) cone type and (iii) fusiform type.

### Statistical Analysis

All data were evaluated using Statistical Package for the Social Sciences software (version 26.0, IBM, Chicago, IL). The comparison of normally distributed quantitative data (the largest diameter of the femoral tunnel) between the two groups was conducted using the Student *t*‐test. The Wilcoxon rank sum test was used to contrast subjective functional scores at baseline and the last follow‐up visit within each group. The Mann–Whitney *U*‐test was undertaken to identify the disparity between the two groups without a normal distribution (subjective functional scores, age at surgery, body mass index (BMI), time from injury to surgery, follow‐up time, side‐to‐side difference of KT‐1000, and the diameter of the entrance of the femoral tunnel and 1 cm proximal to the entrance of the femoral tunnel). The χ^2^‐test (gender) and Fisher's exact test (meniscal involvement, lachman test, complications, and bone tunnel morphology) were used for categorical variables between the two groups. The significance level was set at *p* < 0.05. The sample size and power calculation were performed by an independent statistician using the G*Power program (version 3.1, Heinrich‐Heine‐Universität Düsseldorf). In this study, *a priori* power analysis with a power of 0.8, effect size of 0.8, and significance level of 0.05 indicated that a total of 26 patients were required for each group to detect statistically significant differences in the IKDC scores or bone tunnel enlargement.

## Results

### Baseline Patient Characteristics

Due to the relatively explicit exclusion criteria, we exempted 199 patients, and a total of 102 patients (68 men and 34 women) were recruited in this study with an average age of 32.94 ± 12.42 years at the time of surgery. Among these patients, 39 cases were included in the EB group, and 63 cases were included in the EBIS group. There were no significant differences between the two groups in age, gender, BMI, and the duration time from injury to surgery. Insignificant differences were also found in meniscal involvement, with subsequent need for repair or partial meniscectomy at the time of ACLR (Table 1). The mean follow‐up duration time was 5.05 ± 3.29 years in the EB group and 5.49 ± 3.10 years in the EBIS group. As depicted in Table 2, no statistical differences were discovered regarding the baseline functional scores (IKDC, Lysholmand Tegner activity level scale) and the outcomes of the preoperative physical exam (Lachman test).

**TABLE 1:** Patient demographics

Variable	EB group (n = 39)	EBIS group (n = 63)	Statistic value	*p‐*value
Sex, n (male/female)	24/15	44/19	0.747 (*χ*2)	0.517^b^
Age at surgery (years)^a^	31.82 ± 12.88	33.63 ± 12.18	−0.882 (*Z*)	0.378^c^
BMI^a^	24.87 ± 3.13	24.92 ± 6.83	−0.462 (*Z*)	0.644^c^
Meniscal involvement, n (%)			3.963 (*χ*2)	0.140^d^
No meniscal tear	10 (25.64%)	9 (14.29%)		
Meniscal tear need partial meniscectomy	10 (25.64%)	11 (17.46%)		
Meniscal tear need meniscal repair	19 (48.72%)	43 (68.25%)		
Time from injury to surgery (months)^a^	7.51 ± 20.91	6.08 ± 16.89	−0.207 (*Z*)	0.836^c^

Abbreviations: BMI, body mass index; EB, EndoButton‐continuous loop; EBIS, EndoButton‐continuous loop combined with a bioabsorbable interference screw.

^a^ Values are presented as mean ± SD.

^b^ χ^2^‐test.

^c^ Mann–Whitney *U*‐test.

^d^ Fisher exact test.

**TABLE 2:** Preoperative results of functional scores and clinical evaluation

Variable	EB group (n = 39)	EBIS group (n = 63)	Statistic value	*p‐*value
Baseline IKDC score^a^	67.29 ± 15.30	68.33 ± 12.40	−0.062 (Z)	0.951^b^
Baseline Lysholm score^a^	68.99 ± 9.56	69.67 ± 8.91	−0.277 (Z)	0.782^b^
Baseline Tegner activity scale^a^	2.62 ± 1.46	2.92 ± 1.60	−0.669 (Z)	0.504^b^
Lachman test, n (%)			0.031 (χ2)	1.000^c^
Positive (+)	38 (97.44%)	61 (96.83%)		
Negative (−)	1 (2.56%)	2 (3.17%)		

Abbreviations: EB, EndoButton‐continuous loop; EBIS, EndoButton‐continuous loop combined with a bioabsorbable interference screw; IIKDC, International Knee Documentation Committee.

^a^ Values are presented as mean ± SD.

^b^ Mann–Whitney *U*‐test.

^c^ Fisher exact test.

### Patient‐Reported Outcome Scores

Both groups demonstrated significant improvement in postoperative functional scores on the IKDC, Lysholm, and Tegner activity level scale compared with preoperative results (Table 3). However, there were no considerable distinctions in postoperative functional outcomes between the two groups at the endpoint follow‐up, although the data for KOOS‐Quality of Life in the EBIS group were slightly enhanced compared to those in the EB group (Table 4).

**TABLE 3:** Comparison of preoperative and postoperative results of functional scores and clinical evaluation within groups

Variable	EB group (n = 39)				EBIS group (n = 63)			
	Preoperation	Postoperation	Statistic value	*p‐*value	Preoperation	Postoperation	Statistic value	*p‐*value
IKDC^a^	67.29 ± 15.30	87.53 ± 12.68	−5.444 (Z)	<0.001^b^	68.33 ± 12.40	89.59 ± 8.54	−6.901 (Z)	<0.001^b^
Lysholm^a^	68.99 ± 9.56	90.36 ± 9.62	−5.408 (Z)	<0.001^b^	69.67 ± 8.91	90.30 ± 8.22	−6.956 (Z)	<0.001^b^
Tegner activity scale^a^	2.62 ± 1.46	5.46 ± 1.65	−5.446 (Z)	<0.001^b^	2.92 ± 1.60	5.68 ± 1.59	−6.910 (Z)	<0.001^b^
Lachman test, n (%)			66.505 (χ2)	<0.001^c^			110.508 (χ2)	<0.001^c^
Positive (+)	38 (97.44%)	2 (5.13%)			61 (96.83%)	2 (3.17%)		
Negative (−)	1 (2.56%)	37 (94.87%)			2 (3.17%)	61 (96.83%)		

Abbreviations: EB, EndoButton‐continuous loop; EBIS, EndoButton‐continuous loop combined with a bioabsorbable interference screw; IKDC, International Knee Documentation Committee.

^a^ Values are presented as mean ± SD.

^b^ Wilcoxon rank sum test.

^c^ Fisher exact test.

**TABLE 4:** Patient‐reported outcomes and clinical evaluation between groups at the endpoint follow‐up

Variable	EB group (n = 39)	EBIS group (n = 63)	Statistic value	*p‐*value
Patient‐reported subjective scores^a^				
IKDC	87.53 ± 12.68	89.59 ± 8.54	−0.383 (Z)	0.702^b^
Lysholm	90.36 ± 9.62	90.30 ± 8.22	−0.385 (Z)	0.700^b^
Tegner activity scale	5.46 ± 1.65	5.68 ± 1.59	−0.568 (Z)	0.570^b^
KOOS—Quality of life	81.65 ± 16.88	86.66 ± 13.57	−1.556 (Z)	0.120^b^
Lachman test, n (%)			0.244 (χ2)	0.636^c^
Positive (+)	2 (5.13%)	2 (3.17%)		
Negative (−)	37 (94.87%)	61 (96.83%)		
KT‐1000 (134N), STS, mm^a^	1.63 ± 1.39	1.56 ± 1.12	−0.083 (Z)	0.934^b^
Complications, n (%)				
Deep vein thrombosis	1 (2.56%)	0 (0%)	1.631 χ2)	0.382^c^
Secondary meniscal lesions	1 (2.56%)	1 (1.59%)	0.120 (χ2)	1.000^c^
Knee stiffness	0 (0%)	2 (3.17%)	1.263 (χ2)	0.523^c^
Anterior knee pain	1 (2.56%)	5 (7.94%)	1.256 (χ2)	0.403^c^
Graft failure	2 (5.13%)	1 (1.59%)	1.058 (χ2)	0.556^c^
Contralateral ACL injury	0 (0%)	2 (3.17%)	1.263 (χ2)	0.523^c^

Abbreviations: EB, EndoButton‐continuous loop; EBIS, EndoButton‐continuous loop combined with a bioabsorbable interference screw; IKDC, International Knee Documentation Committee; STS, side‐to‐side.

^a^ Values are presented as mean ± SD.

^b^ Mann–Whitney *U*‐test.

^c^ Fisher exact test.

### Clinical Evaluation

As the results of the Lachman test presented in Tables 3 and 4, the postoperative stability of the knee joint in both groups was considerably better than that before surgery, but there was no significant disparity between the two groups at the endpoint follow‐up. The postoperative mean KT‐1000 side‐to‐side difference in the EB group was 1.63 ± 1.39 mm, while the postoperative mean KT‐1000 side‐to‐side difference in the EBIS group was 1.56 ± 1.12 mm (*p* = 0.934).

### Postoperative Complications

Only one patient in the EB group had a deep vein thrombosis 1 week following surgery. The patient remained in the hospital and received oral medication. One patient in each group had a secondary meniscal injury postoperatively and needed arthroscopic surgery. Two patients in the EBIS group revealed postoperative knee stiffness during follow‐up. One patient had anterior knee pain in the EB group compared to five patients in the EBIS group. There were two graft failures (one due to the advancement of osteoarthritis and another due to unsatisfactory postoperative outcomes because of the inappropriate bone tunnel position shown in MRI images) in the EB group and one (due to sports injury) in the EBIS group. There were two contralateral ACL injuries in the EBIS group (all due to sports). There was no statistical difference in the occurrence of each complication between the two groups.

### MRI Assessment

All patients included in this study (39 patients in the EB group and 63 patients in the EBIS group) had MRI images obtained at two time points simultaneously (two time points: at least 2 years and within 2 weeks after surgery). As outlined in Table 5, unlike the EBIS group, the EB group displayed slightly increased diameters of BTE at all three measurement sites. However, no significant difference was found between the two groups (*p* = 0.198 at the entrance of the femoral tunnel, *p* = 0.318 at the position of 1 cm proximal to the entrance of the femoral tunnel, and *p* = 0.123 at the largest diameter of the femoral tunnel). The most common shape of BTE following surgery in both groups was the line type (58.97% in the EB group *vs*. 66.66% in the EBIS group). The proportion of conical bone tunnel morphology in the EB group (15.38%) was higher than that in the EBIS group (7.94%), while the quantity of fusiform bone tunnel morphology was comparable in the EB group (25.64%) and the EBIS group (25.40%). There was no significant variance in the distribution of BTE morphology between groups.

**TABLE 5:** Measurement of the femoral tunnel enlargement in magnetic resonance images.

Enlargement of the bone tunnel	EB group (n = 39)	EBIS group (n = 63)	Statistic value	*p‐*value
The entrance of the femoral tunnel^a^ (mm)	2.72 ± 1.89	2.18 ± 1.84	−1.288 (Z)	0.198^c^
1 cm proximal to the entrance of the femoral tunnel^a^ (mm)	2.77 ± 1.60	2.42 ± 1.81	−0.999 (Z)	0.318^c^
The largest diameter of the femoral tunnel^a^ (mm)	3.25 ± 1.69	2.69 ± 1.81	1.556 (T)	0.123^b^
Bone tunnel morphology (%)			1.463 (χ2)	0.505^d^
Line type	23 (58.97%)	42 (66.66%)		
Cone type	6 (15.38%)	5 (7.94%)		
Fusiform type	10 (25.64%)	16 (25.40%)		

Abbreviations: EB, EndoButton‐continuous loop; EBIS, EndoButton‐continuous loop combined with a bioabsorbable interference screw.

^a^ Values are presented as mean ± SD.

^b^ Student's *t*‐test.

^c^ Mann–Whitney *U*‐test.

^d^ Fisher exact test.

## Discussion

The principal finding of the present study was that the clinical outcome of ACLR with quadruple hamstring autograft was not influenced by the application of different femoral fixation methods. Meanwhile, concerning the development of BTE, although the mean diameters of BTE in the EB group were slightly higher than those in the EBIS group, no statistically significant difference was found between the hybrid fixation and suspension fixation on the femoral side.

### Preoperative Baseline Data

To inhibit the influence of confounding factors so that we could simply contrast the effects of different femoral fixation methods on the enlargement of the bone tunnel and the clinical results, we unified the type of tendon graft, the position of the bone tunnel, the type of tibial fixation, and the rehabilitation protocol. Some recent studies have indicated that the time from injury to surgery will also affect the enlargement of the bone tunnel as well as the risk of revision ACLR.^29^, ^30^ Fortunately, in our analysis, there was no significant difference in the time from injury to surgery between the two groups. Cristiani *et al*.^29^ further described that there was no relationship between medial or lateral meniscus resection or repair at the time of primary ACLR and postoperative clinical outcomes. Because no statistical differences were observed in the baseline data of all included patients, we presume that our findings were identified within two homogeneous groups exhibiting comparable preoperative activity levels and functional scores.

### Controversy over Different Fixation Methods on the Femoral Side

Exploring the micromotion between the graft and bone tunnel plays a crucial role in the topic of BTE after ACLR.^9^ Graft–tunnel motion indicates the longitudinal (bungee effect) and transverse (windshield wiper effect) motion of the graft within the bone tunnel and can occur with several fixation techniques.^31^, ^32^ Many investigators have revealed that a fixation distant from the joint line, for example using the EndoButton, might allow more graft–tunnel interface micromotion and influence the phenomena of the bungee and windshield wiper effects, which will facilitate increased BTE.^33^, ^34^ Other researchers also found that fixation at the level of the joint line (e.g., using a bioabsorbable interference screw) might mitigate the above‐described effects.^13^, ^35^ However, inconsistent results have been discovered in the literature. Mermerkaya *et al*.^6^ indicated that tunnel widening after ACLR was not lowered when interference screw fixation was applied. In a recent prospective randomized study, the investigators discovered that the interference screw fixation group had significantly greater tunnel enlargement in anteromedial tunnels than the EndoButton fixation group.^36^ In conjunction with some inherent disadvantages of bioabsorbable interference screws, which include inflammatory response, bone resorption, loosening, and cyst formation, the application of bioabsorbable interference screws alone for femoral graft fixation is decreasing.^37^, ^38^ To explore a superior fixation method on the femoral side, we innovatively developed a fixation method with a combination of EndoButton and the bioabsorbable interference screw.

### Comparative Study of Postoperative Bone Tunnel Enlargement

Theoretically, the hybrid femoral fixation could lower the possibility of graft migration. Furthermore, the bungee effect and the windshield wiper effect might be lowered owing to the barrier created by the surrounding interference screw. Contrary to our hypothesis, there were no statistically significant differences in the BTE diameters between the two groups more than 2 years after ACLR, as determined from MRI images. We contrasted three areas of the femoral tunnel between the two groups to more adequately reflect the enlargement of the tunnel after surgery and to examine the shape of the bone tunnel. By measurement, the mean diameter of the enlarged femoral tunnel in the EBIS group was smaller than that in the EB group, which might be linked to the addition of a bioabsorbable interference screw in the femoral tunnel near the joint line. However, we could not infer that hybrid femoral fixation indicated less BTE than EndoButton‐only fixation after ACLR with hamstring autograft because the *p*‐value was >0.05. Our conclusions were comparable to the study of Ma, who discovered no considerable difference in BTE between patients who had hamstring ACLR with a bioabsorbable interference screw fixation and EndoButton fixation.^39^ Consistent with our findings, Höher *et al*.^31^ outlined three morphologies of the enlarged bone tunnels: “cone type,” “line type,” and “cavity type.” We found that the most common femoral widening in both the EB and EBIS groups was the line type. That implied that the femoral tunnel was more often enlarged uniformly from the entrance to the distal end of the tunnel, which was irregular with the prior report that the cone type was the most prevalent shape of the tunnel enlargement.^28^ In the EB group, the proportion of femoral bone tunnels with cone shape (15.38%) was a bit higher than that (7.94%) in the EBIS group. It can be determined by the fixation placed further away from the joint line, which is most noticeably linked to the windshield wiper effect. A similar observation indicated that the bone tunnel usually expanded in the direction of joint space and the least evident location was around the fixation points.^40^

### Analysis of Clinical Outcome in Two Patient Groups Post‐Surgery

The results of this research indicated that both cortical suspension and hybrid femoral fixation techniques in hamstring ACLR significantly improved postoperative outcomes, with no significant differences between the two methods in terms of clinical evaluations, postoperative complications, and patient‐reported outcome scores. Our outcomes were comparable to those of many other studies that stated BTE had no evident correlation with clinical outcomes.^41^, ^42^ The mean side‐to‐side difference between the ACL‐reconstructed knee and the contralateral healthy knee was less than 3 mm (knee laxity threshold of KT‐1000) at the last follow‐up point after surgery in both groups. Therefore, both femoral fixation methods provided sufficient stability and strength for patients. In a systematic review of high‐quality studies, the authors also showed that comparable outcomes and time to return to sports were found in ACLR patients with intra‐tunnel or extra‐tunnel fixation.^43^ Furthermore, despite our low postoperative complication rate, we had one case where graft failure occurred 1 year after surgery due to a deviation of the bone tunnel position. Thus, placement of the graft in the right place with an adequate method might be more essential than what is used for fixation. Based on our research findings, it can be inferred that adding a bioabsorbable interference screw for hybrid fixation on the femoral side will inevitably prolong operation times and increase surgical costs, but it does not improve postoperative functional scores or reduce the incidence of complications. Therefore, we do not recommend the use of hybrid fixation on the femoral side for the purpose of reducing postoperative BTE in ACLR.

### Timing and Methods for Postoperative Imaging Studies on Bone Tunnel Enlargement

Regarding the BTE time frame after surgery, many studies have proposed that bone tunnel widening had stabilized by 6 months following surgery.^44^ Bhullar *et al*. reported that the time needed for MRI diagnosis of BTE was approximately 17.7 months.^45^ Therefore, our shortest MRI follow‐up period was 2 years, and it might thus be anticipated that all tunnel diameters had attained their maximum by the time all images were measured by the investigator. For the accuracy of pre‐post MRI image comparison, we analyzed the MRI images of the femoral tunnel within 2 weeks after ACLR instead of using the diameter of the drill during the surgery as the original data.

Plain radiographs, computed tomography (CT), and MRI can all be used to evaluate the position and enlargement of bone tunnels after ACLR.^46^ Due to the superiority of 3D analysis, reliable imaging modality and more precise imaging to assess the bone tunnel boundaries, some authors recommend CT for postoperative evaluation of ACLR.^47^ However, MRI is also an effective and useful assessment tool for evaluating the BTE due to the improvement of MRI imaging resolution (3 Tesla) and the fact that MRI can detect more information about the status of the reconstructed ligament than CT.^36^, ^48^ Furthermore, while CT is the most reliable for assessing BTE compared to X‐rays and MRI, its additional radiation risks prompt some authors to advocate for MRI as an alternative for bone tunnels evaluation.^45^ In the clinical practice of this study, we also found that MRI can clearly display the femoral bone tunnel and complete the measurement of BTE data.

### Limitations and Strengths

This research has several limitations. First, since this is a randomized retrospective case–control study, selection bias cannot be overlooked. Second, the high number of patients lost to follow‐up led to a relatively small group. Although our longest follow‐up period was over 11 years, 102 patients were eventually recruited. Third, there might be measurement bias in measuring the diameter of the bone tunnel in MRI images because of the selection of the MRI sequence. Meanwhile, further research is needed to determine the actual advantages between MRI and CT examinations in studying BTE. Furthermore, the size of the bioabsorbable interference screw on the femoral side was the same regardless of the size of the femoral bone tunnel in this study. We made this choice because during the actual surgical procedure, we found that a 6‐mm screw was already tight when screwed in, and using a screw with the same diameter as the femoral tunnel might risk blowing out the posterior wall of the femoral tunnel. This mismatch might also have a subtle effect on the assessment of BTE.

Despite the mentioned limitations, this study is among the few to evaluate BTE and clinical outcome in ACLR, comparing cortical suspension and hybrid femoral fixation. Through a detailed comparison of follow‐up results, the study holds significant implications for clinical practice.

## Conclusion

Adding a bioabsorbable interference screw near the joint line in the femoral tunnel was not superior to EndoButton‐only fixation on the femoral side in hamstring ACLR. No significant difference was found in the BTE and clinical outcome between cortical suspension and hybrid femoral fixation in ACLR using hamstring autograft.

## Conflict of Interest Statement

The authors declare that they have no conflict of interest.

## Ethical Statement

This study was established and authorized by the institutional ethics review board of Zhongda Hospital, Southeast University (2021ZDSYLL345‐P01).

## Author Contributions

Yucheng Lin and Lu Zhang contributed equally to this work. Yucheng Lin and Lu Zhang: study design, data collection, data analysis, data interpretation, manuscript drafting, and critical revision. Sinuo Shen and Yuzhi Chen: statistical analysis. Li Xu and Mingliang Ji: MRI images analysis and measurement. Yudong Guo and Jinan Wei: data collection and data analysis. Yonggang Li and Xiaotao Wu: data interpretation and manuscript drafting. Jun Lu: study design, data analysis, data interpretation, and critical revision. All authors have read and approved the manuscript.

## Data Availability

The data and materials are available from the medical records department of Zhongda Hospital. The datasets used and analyzed during the study are available from the corresponding author on reasonable request.
